# Supplementary material for: Resolving the full spectrum of human genome variation using Linked-Reads
Source: Genome Res. 2019 Apr;29(4):635–45. doi: 10.1101/gr.234443.118 (PMC6442396; doi:10.1101/gr.234443.118)
Supplement: Supplemental Material [file supp_gr.234443.118_Supplemental_longranger-2.2.zip › longranger-2.2.tar.bz2!/lr2.2-public/lib/go/src/code.google.com/vitess/vitess.io/_includes/left-nav-menu.html]

- #### Overview

  - What is Vitess
  - Scaling MySQL with Vitess
  - Key Concepts
- #### Getting Started

  - Run Vitess on Kubernetes
    - Custom Docker Build
  - Run Vitess Locally
- #### User Guide

  - Introduction- Client Libraries- Backing Up Data- Reparenting
        - Schema Management
        - VSchema Guide
        - Schema Swap (Tutorial)
        - Sharding
          - Horizontal Sharding (Tutorial, automated)
          - Horizontal Sharding (Tutorial, manual)
          - Sharding in Kubernetes (Tutorial, automated)
          - Sharding in Kubernetes (Tutorial, manual)
        - Vitess Sequences
        - MySQL Server Protocol
        - Vitess and Replication
        - Update Stream
        - Row Based Replication
        - Topology Service
        - Transport Security Model
        - Launching
          - Scalability Philosophy
          - Production Planning
          - Server Configuration
          - 2PC Guide
          - Troubleshooting
        - Upgrading
- #### Reference Guides

  - Vitess API- vtctl Commands
- #### Other Resources

  - Presentations- Blog- Roadmap
- #### Contributing

  - Contributing to Vitess- GitHub Workflow- Code Reviews
